# Haploinsufficiency of *Cyfip1* Produces Fragile X-Like Phenotypes in Mice

**DOI:** 10.1371/journal.pone.0042422

**Published:** 2012-08-10

**Authors:** Ozlem Bozdagi, Takeshi Sakurai, Nathan Dorr, Marion Pilorge, Nagahide Takahashi, Joseph D. Buxbaum

**Affiliations:** 1 Seaver Autism Center for Research and Treatment, Mount Sinai School of Medicine, New York, New York, United States of America; 2 Department of Psychiatry, Mount Sinai School of Medicine, New York, New York, United States of America; 3 Department of Neuroscience, Mount Sinai School of Medicine, New York, New York, United States of America; 4 Department of Genetics and Genomic Sciences, Mount Sinai School of Medicine, New York, New York, United States of America; Johns Hopkins, United States of America

## Abstract

**Background:**

Copy number variation (CNV) at the 15q11.2 region, which includes a gene that codes for CYFIP1 (cytoplasmic FMR1 interacting protein 1), has been implicated in autism, intellectual disability and additional neuropsychiatric phenotypes. In the current study we studied the function of Cyfip1 in synaptic physiology and behavior, using mice with a disruption of the *Cyfip1* gene.

**Methodology/Principal Findings:**

We observed that in *Cyfip1* heterozygous mice metabotropic glutamate receptor (mGluR)-dependent long-term depression (LTD) induced by paired-pulse low frequency stimulation (PP-LFS) was significantly increased in comparison to wildtype mice. In addition, mGluR-LTD was not affected in the presence of protein synthesis inhibitor in the *Cyfip1* heterozygous mice, while the same treatment inhibited LTD in wildtype littermate controls. mGluR-agonist (RS)-3,5-dihydroxyphenylglycine (DHPG)-induced LTD was also significantly increased in hippocampal slices from *Cyfip1* heterozygous mice and again showed independence from protein synthesis only in the heterozygous animals. Furthermore, we observed that the mammalian Target of Rapamycin (mTOR) inhibitor rapamycin was only effective at reducing mGluR-LTD in wildtype animals. Behaviorally, *Cyfip1* heterozygous mice showed enhanced extinction of inhibitory avoidance. Application of both mGluR5 and mGluR1 antagonist to slices from *Cyfip1* heterozygous mice reversed the increase in DHPG-induced LTD in these mice.

**Conclusions/Significance:**

These results demonstrate that haploinsufficiency of *Cyfip1* mimics key aspects of the phenotype of *Fmr1* knockout mice and are consistent with the hypothesis that these effects are mediated by interaction of Cyfip1 and Fmrp in regulating activity-dependent translation. The data provide support for a model where *CYFIP1* haploinsufficiency in patients results in intermediate phenotypes increasing risk for neuropsychiatric disorders.

## Introduction

CNVs in the 15q11.2 (BP1–BP2) region represent replicated risk factors for schizophrenia, epilepsy, intellectual disability, developmental delay, and autism [1], [2], [3], [4], [5]. For example, in two large studies of schizophrenia recurrent CNVs were identified which involved the 15q11.2 region that were associated with increased risk [6], [7]. The CNV includes a minimal 0.3 Mb region that encompasses five refseq genes (*TUBGCP5*, *CYFIP1*, *NIPA2*, *NIPA1,* and *WHAMML1,* see [8]) and increases risk for schizophrenia by 2–4 fold. This interval has already been of interest in psychiatric disorders because of its involvement in autism spectrum disorders (ASD) involving duplications of 15q11-q13 and Prader-Willi and Angelman syndromes [9]. Type I deletions of Prader-Willi and Angelman syndromes, which include this interval, have been associated with more severe manifestations, as compared to deletions (type II) that do not include this interval, including greater severity of ASD features [10], [11], [12], [13]. Several studies indicate that this same region increases risk for developmental disorders including ASD, likely in the presence of other genetic risk factors [14], [15], [16], [17]. In the largest study to date, involving over 15,000 patient samples, deletion of the 15q11.2 region was very strongly associated with developmental delays including ASD, with incomplete penetrance [16].

*CYFIP1*, a gene in this region, codes for a protein that binds the Fragile X protein FMRP, and is therefore a candidate gene for psychiatric phenotypes. Fragile X syndrome (FXS) is the most common inherited form of intellectual disability, is frequently associated with co-morbid ASD, and is most commonly caused by transcriptional silencing of the *FMR1* gene [18]. The gene product of *FMR1* is fragile X mental retardation protein (FMRP). CYFIP1 was identified as one of two highly conserved cytoplasmic FMR1 interacting proteins [19]. In addition to its interaction with FMRP, CYFIP1 was also shown to interact with the small GTPase Rac1 [20]. Both FMRP and Rac1 have been shown to be involved in neuronal and synaptic function. FMRP is regulated in response to mGluR activation [21], [22], [23] and *Fmr1* knockout mice show increased mGluR-dependent LTD (mGluR-LTD) [24]. FMRP has been shown to be involved in negatively regulating translation in synapses and this negative regulation can be removed as a result of neuronal activity [18]. Disruption of FMRP in FXS hence results in increased translation of synaptic proteins, which in turn can lead to down-regulation of the alpha-amino-3-hydroxy-5-methyl-4-isoxazolepropionic acid (AMPA) receptors, and lead to disruption of normal synaptic plasticity. Enhanced mGluR-LTD in the absence of FMRP has given rise to the mGluR hypothesis of FXS; higher hippocampal (mGluR-dependent) LTD in *Fmr1* knockout mice no longer shows a requirement for protein synthesis (as the normal control on protein synthesis mediated by FMRP is lost). Recently, it has been shown that CYFIP1 can directly bind to the translation initiation factor eIF4E and, like FMRP, negatively regulates FMRP target mRNAs [25]. Stimulation of neurons was shown to cause the dissociation of CYFIP1 from eIF4E at synapses, resulting in protein synthesis, thus providing a mechanism for the activity-dependent regulation of translation seen with FMRP and CYFIP1. The association of CYFIP1 with FMRP, the latter a protein directly implicated in neurodevelopment and psychiatric disorders, makes CYFIP1 a very compelling candidate for an important role in the phenotypes associated with CNVs in the 15q11.2 region [26], [27], [28], [29]. At the same time haploinsufficiency of any or all of the additional genes in the region may also have an additional contributory role.

In order to characterize the functions of *CYFIP1* as related to neuropsychiatric phenotypes we developed mice with a disruption in *Cyfip1*. Similar to what has been observed in *Fmr1* knockout mice, *Cyfip1* heterozygous mice expressed enhanced mGluR-LTD, which was resistant to protein synthesis inhibition. LTD in the *Cyfip1* heterozygous mice was insensitive to inhibition of mTOR pathway, in contrast to what was observed in wildtype littermates. Furthermore, *Cyfip1* heterozygous mice exhibited enhanced extinction of inhibitory avoidance. Finally, exposure of hippocampal slices to mGluR antagonists reversed the increase in mGluR-LTD in *Cyfip1* knockout mice. The remarkable overlap in phenotype between *Fmr1* knockout mice and *Cyfip1* heterozygous mice is consistent with a model in which *CYFIP1* haploinsufficiency results in intermediate phenotypes increasing risk for neuropsychiatric disorders.

## Results

### Generation and Validation of Mice with a Disruption in the *Cyfip1* Gene

**Figure 1:**
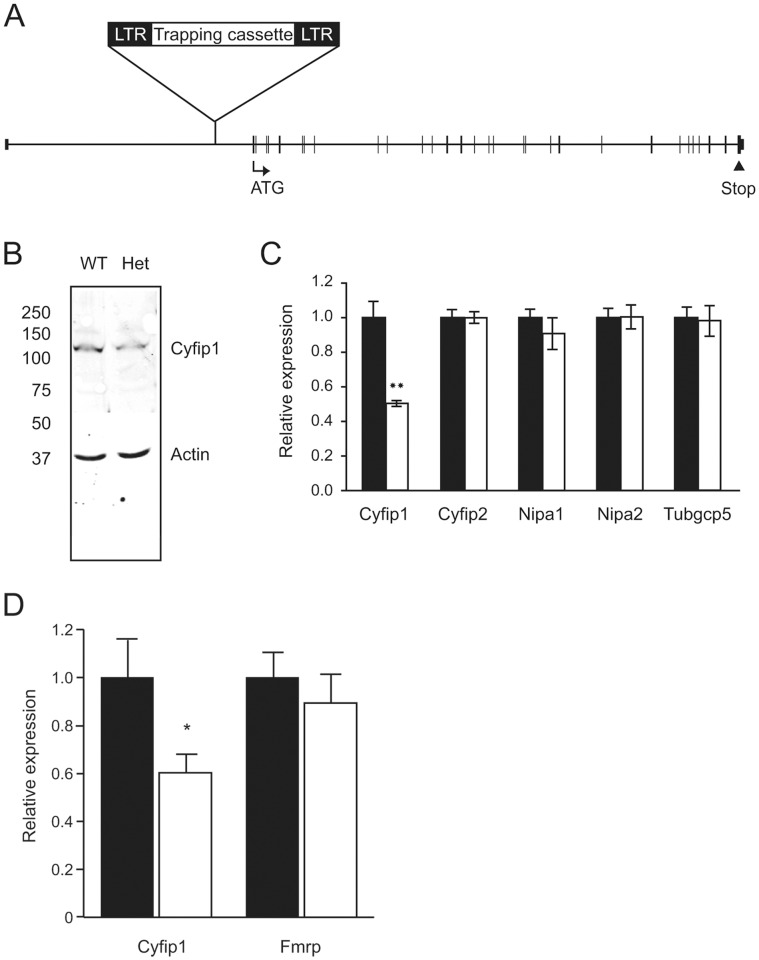
Generation and characterization of a mouse with disruption of the *Cyfip1* gene. (**A**) The genomic structure of *Cyfip1* is shown to scale with larger horizontal boxes representing exons, and the first (ATG) and last (Stop) coding exons indicated. The diagram shows the site of the gene-trap insertion (identified as an LTR-flanked Trapping casette) in intron 1 (5′ to the first coding exon), in order to generate mice with a disruption of the *Cyfip1* gene. (**B**) Synaptoneurosome preparations from wildtype (Wt) and *Cyfip1* heterozygous (Het) mice were subjected to quantitative immunoblotting with an antibody to Cyfip1, with actin as a reference protein. The migration of molecular weight markers is shown on the left (in kDa). (**C**) Brain mRNA from wildtype (black bars) and *Cyfip1* heterozygous (white bars) mice were subjected to qPCR for the indicated genes. (**D**) Quantification of Cyfip1 and Fmrp by immunoblotting of extracts from wildtype (black bars) and heterozygous (white bars) mice. *, P<0.05; **, P = 0.004.

To directly test whether *Cyfip1* expression is required in development and synaptic function, we made use of gene-trapped embryonic stem (ES) cells to develop mice with a disruption in *Cyfip1* (Fig. 1A). Despite numerous attempts, we never recovered mice with a disruption of both copies of *Cyfip1* (knockouts), and even at embryonic days 4 and 5 there was no evidence for knockout embryos. These results indicate that *Cyfip1* is necessary for early embryonic development. We did obtain mice with a disruption of a single copy of *Cyfip1* (heterozygotes) at expected ratios, when crossing heterozygotes *inter se* or crossing heterozygotes with wildtype animals.

To confirm reduced expression of Cyfip1, we made use of immunoblotting using cortices from 4 weeks old mice and observed a ∼40% reduction in expression of Cyfip1 in heterozygotes, as compared to wildtype littermates (Figs. 1B, 1D). We also measured gene expression by quantitative PCR (qPCR), and found that *Cyfip1* mRNA is reduced by 50% (Fig. 1C). There was no compensatory change in *Cyfip2*, nor was there any change in expression from other genes that flank *Cyfip1* (*Tubgpc5*, *Nipa1*, *Nipa2*), which might have been affected by the gene trap (Fig. 1C). Furthermore, we did not detect potential compensatory changes in levels of Fmrp (Fig. 1D).

Developmental milestones, assessed by physical development, motor development, and reflexes, were normal in *Cyfip1* heterozygote mice. The assessments included monitoring the development of cliff avoidance, forelimb placing, vibrissa placing, visual placing, auditory startle, tactile startle and toe pinch. Fear-induced freezing was also measured. There was no difference between wildtype and *Cyfip1* heterozygous mice in any of these measures. Altogether, developmental milestones in the heterozygotes were within normal limits, making them appropriate for further, detailed studies.

### Increased mGluR-dependent Long-term Depression in *Cyfip1* Heterozygotes

**Figure 2:**
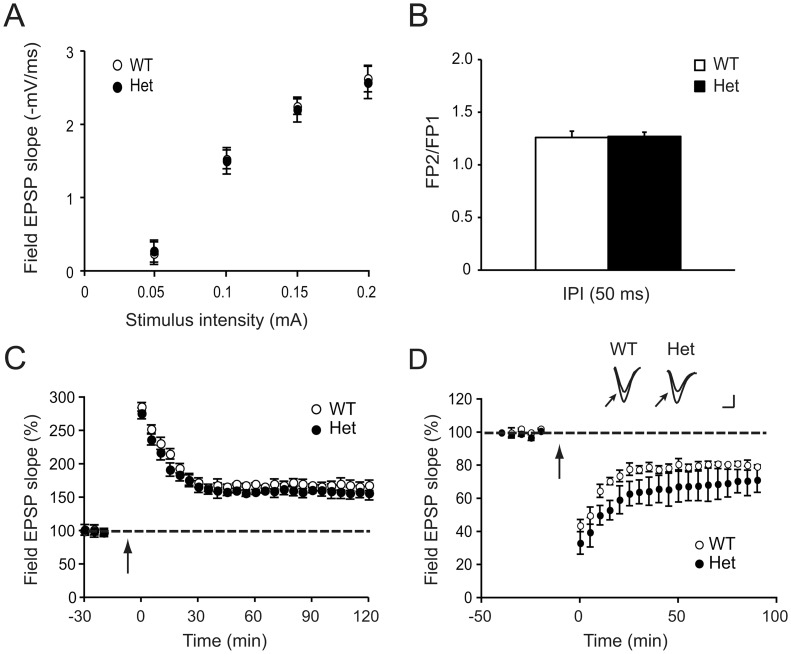
Basal synaptic properties and long-term potentiation are normal but long-term depression is enhanced in *Cyfip1* heterozygotes. (**A**) Hippocampal slices from 4–6 weeks old wildtype (WT) or Cyfip1 heterozygous (Het) mice were analyzed for baseline synaptic properties, determined by input/output function, representing the relationship between stimulus intensity and the size of the field EPSP slope. (**B**) Paired-pulse facilitation in the Schaffer collateral-commissural pathway is not different between genotypes over the test interpulse interval of 50 ms. (**C**) HFS-induced LTP was not significantly different between wildtype (WT) or *Cyfip1* heterozygous (Het) mice. (**D**) PP-LFS-induced LTD in *Cyfip1* heterozygous mice was significantly increased. Inset: Representative EPSP traces recorded before stimulation (arrow) or 60 min after stimulation in wildtype and heterozygous animals (scale: 10 ms and 0.5 mV).

There have been extensive studies of the effects of loss of FMRP on electrophysiological properties in the hippocampus, particularly as pertains to synaptic plasticity. The association of FMRP with CYFIP1 supported similar analyses in the *Cyfip1* heterozygotes. We first examined basal synaptic transmission in the Schaffer collateral pathway in hippocampal slices, determined by input/output analyses and paired-pulse facilitation, to study the effect of Cyfip1 on presynaptic release probability and short-term synaptic plasticity. We found no significant differences between genotypes in input/output function (Fig. 2A) or in paired-pulse ratio over the test interpulse interval of 50 ms (1.26±0.06 and 1.27±0.04, in wildtype and heterozygous mice, respectively, using 6 mice per genotype, p = 0.4, Student’s t-test) (Fig. 2B). Together these results indicate that neither basal synaptic transmission nor the efficiency of neurotransmitter release is altered in *Cyfip1* heterozygotes.

Long-term potentiation (LTP) is an important measure of synaptic plasticity. We induced LTP by high frequency stimulation consisting of four trains of 100 Hz for 1 second, separated by 5 minutes, a protocol which induces a protein synthesis-dependent form of LTP (Fig. 2C). The induction and maintenance of LTP were not significantly different between hippocampal slices from wildtype and heterozygous mice over the 120 min time course after tetanic stimulation (average percentage of baseline 120 min after tetanus: wildtype, 168.6±9.4%; heterozygote, 159.8±11.4%; n = 6 slices, 4 mice per group; F (1,10) = 1.1 p = 0.23). In another set of experiments we further tested LTP induced by threshold levels of theta-burst afferent stimulation, which also did not demonstrate differences between genotypes (average percentage of baseline 30 min after tetanus: wildtype, 122.1±4.2%; heterozygote, 116.8±8.9%; n = 6 slices, 4 mice per group; F (1,10) = 2.06 p = 0.12). Finally early phase LTP (E-LTP) was induced with 100 Hz tetanic stimulation, for 1 second. There were no significant differences amongst the two genotypes with this stimulation paradigm (average percentage of baseline 60 min after tetanus: wildtype, 131.7±9%; heterozygote, 130.0±8.1%; F (1,9) = 0.9 p = 0.41).

Metabotropic receptor-dependent long-term depression (mGluR-LTD) is another important measure of synaptic plasticity and one that has been shown to be altered the absence of FMRP. To examine the role of Cyfip1 in mGluR-LTD we recorded field EPSPs at Schaffer collateral-CA1 synapses in acute hippocampal slices. In slices derived from wildtype animals, LTD induced by paired pulse-low frequency stimulation (PP-LFS) resulted in a reduction in field EPSP slope to 79.9±2.4% of baseline, while in slices derived from heterozygous animals the magnitude of the depression was significantly increased, to 67.8±8% of baseline (n = 9 slices, 6 mice for wildtype and 10 slices, 6 mice for heterozygotes; F (1,17) = 5.4 p = 0.03) (Fig. 2D). We then tested the role of Cyfip1 in NMDAR-dependent LTD using 15 min LFS consisting of 900 pulses at 1 Hz (data not shown). There were no significant differences between genotypes with this stimulation paradigm (wildtype, 82.6±3.6%; heterozygote, 79.9±2%; measured 60 min after LFS; n = 7 slices, 4 mice for wildtype and 7 slices, 3 mice for heterozygotes; F (1,12) = 2.15 p = 0.1).

### mGluR-LTD is Independent of Protein Synthesis in *Cyfip1* Heterozygotes

**Figure 3:**
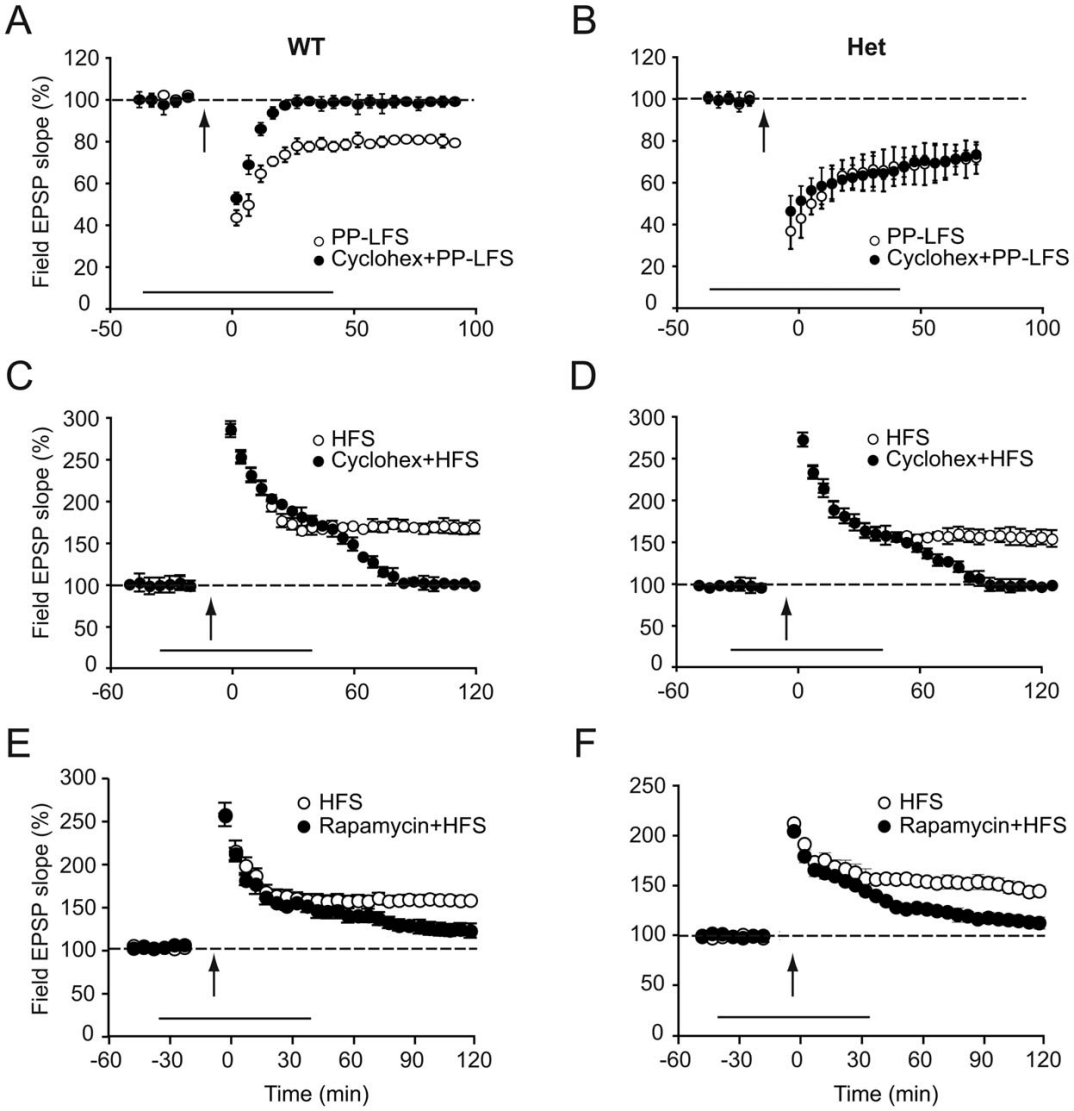
Long-term depression but not long-term potentiation is independent of protein synthesis in *Cyfip1* heterozygotes. (**A, B**) LTD induced by PP-LFS in wild type (**A**) or *Cyfip1* heterozygous (**B**) mice, in the absence (o) or presence (•) of the protein synthesis inhibitor cycloheximide (60 µM). The effect of cycloheximide is significantly different across genotypes. (**C, D**) LTP induced by HFS in wildtype (**C**) or *Cyfip1* heterozygous (**D**) mice, in the absence (o) or presence (•) of cycloheximide. In all panels, the large arrow indicates onset of stimulation. (**E, F**) Rapamycin blocks LTP induced by HFS in wildtype (**E**) and *Cyfip1* heterozygous (**F**) mice, as shown by incubating slices in the absence (o) or presence (•) of the mTOR inhibitor rapamycin (20 nM). Onset of stimulation is indicated by an arrow.

One of the most striking abnormalities in experimental synaptic plasticity observed in the absence of FMRP is that mGluR-LTD is independent of protein synthesis in these animals. As the *Cyfip1* heterozygotes have enhanced LTD, similar to what has been observed in the absence of FMRP, we next compared mGuR-LTD in the absence of presence of the protein synthesis inhibitor cycloheximide. PP-LFS-induced LTD was not affected by the addition of cycloheximide (60 µM) in the *Cyfip1* heterozygous animals, while the same treatment inhibited LTD in the wildtype littermate controls (Figs. 3A, 3B). In the presence of cycloheximide, fEPSP slope in slices from wildtype animals was at 98.05±2.5% of baseline values at 60 minutes after PP-LFS, while in sliced from heterozygous animals the slope was at 68.49±8.9% of baseline (n = 8 slices, 6 mice per group, F (1,17) = 11.7 p = 0.01).

In contrast to the results with LTD, we observed that the inhibition of protein synthesis-dependent LTP with cycloheximide did not differ between genotypes (Figs. 3C, 3D) (measuring at 120 minutes after tetanus, field EPSP slope was 168±9.4% of baseline for wildtype slices, 99±8% for wildtype slices in the presence of cycloheximide, 156.7±11.4% for heterozygous slices, and 100.8±8.2% for heterozygous slices in the presence of cycloheximide). Treatment of the slices with mTOR inhibitor rapamycin also reduced LTP in studies with both the wildtype and heterozygous mice (Figs. 3E, 3F).

### Increased Chemically Induced mGluR-LTD in *Cyfip1* Heterozygous Mice

**Figure 4:**
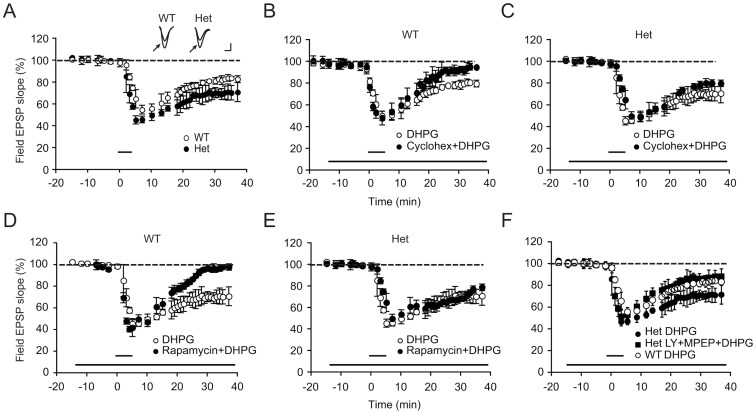
DHPG-induced long-term depression is not dependent on protein synthesis or mammalian Target of Rapamycin in *Cyfip1* heterozygotes. (**A**) LTD was induced by DHPG (50 µM for 5 minutes, indicated by the short horizontal bar) in hippocampal slices from wildtype (Wt) and *Cyfip1* heterozygous (Het) mice. LTD is significantly enhanced in the heterozygotes as compared to wildtype. Inset: Representative EPSP traces recorded before (arrow) or 40 min after DHPG in wildtype and heterozygous animals (scale: 10 ms and 0.5 mV). (**B, C**) LTD was induced with DHPG in wildtype (B) or *Cyfip1* heterozygous (C) mice, in the absence (o) or presence (•) of cycloheximide (Cyclohex, 60 µM, indicated by the long horizontal bar). Cycloheximide significantly inhibited LTD in slices from wildtype but not heterozygous animals. (**D, E**) LTD was induced by DHPG (50 µM, indicated by the short horizontal bar) in hippocampal slices from wildtype (D) or *Cyfip1* heterozygous (E) mice, in the absence (o) or presence (•) of rapamycin (20 nM, indicated by the long horizontal bar). (**F**) LTD was induced by DHPG (50 µM, indicated by the short horizontal bar) in hippocampal slices from wildtype (o) or *Cyfip1* heterozygous (•) mice, the latter in the absence (•) or presence (▪) of both MPEP (10 µM) and LY367385 (indicated by the long horizontal bar). Application of the mGluR1 and mGluR5 antagonists decreased the magnitude of DHPG-induced-LTD in heterozygotes.

The observation that tetanic-stimulation induced LTD, but not LTP, was independent of protein synthesis with a 50% reduction in levels of Cyfip1 was particularly intriguing. To further confirm this finding, we made use of another method to induce LTD, using DHPG, which is an agonist that activates group I mGluRs. This treatment induced a depression in synaptic transmission to 81.2±3% of baseline at 30 min of application in wildtype mice (Fig. 4A). In heterozygous mice, this treatment led to significantly increased LTD (70.5±6% of baseline) as compared to wildtype animals (Fig. 4A, n = 7 slices, 4 mice for wildtype and 8 slices, 4 mice for heterozygotes; F (1,13) = 6.15 p = 0.023). As before, the addition of 60 µM cycloheximide reduced LTD in wildtype, but not *Cyfip1* heterozygous mice (F (1,12) = 1.15 p = 0.2; Figs. 4B, 4C). We next tested mTOR dependency of mGluR-LTD by bath-applying 20 nM rapamycin starting 15 min before DHPG application and continuing through the end of the experiment. We observed that rapamycin treatment reduced mGluR-LTD in wildtype, but not *Cyfip1* heterozygous littermates (F (1,11) = 1.4, p = 0.24; Figs. 4D, 4E).

### Reversal of Enhanced LTD in *Cyfip1* Heterozygotes by mGluR Antagonists

Because mGluR activation is essential for mGluR-LTD induction, we examined the effect of mGluR blockade on DHPG-induced LTD. Slices were incubated in both mGluR1 (LY367385, 100 µM) and mGluR5 (MPEP, 10 µM) antagonists. Bath application of both compounds to slices derived from *Cyfip1* heterozygotes significantly decreased the magnitude of LTD in these slices to control levels (wildtype: 81.2±3% of baseline at 30 minutes of DHPG application, *Cyfip1* heterozygotes, 70.5±6% of baseline, *Cyfip1* heterozygotes in the presence of MPEP and LY367385, 83±5.7% of baseline, F(2,22) = 4.18, p = 0.03) (Fig. 4F).

### Enhanced Extinction of Inhibitory Avoidance in *Cyfip1* Heterozygotes

Anxiety and abnormal social behavior are significant characteristics of several neuropsychiatric disorders in humans. We therefore first examined open field analysis, light dark transition and elevated zero maze in *Cyfip1* heterozygote mice and wildtype littermates and did not observe any differences as a function of genotype (Table S1).

**Figure 5:**
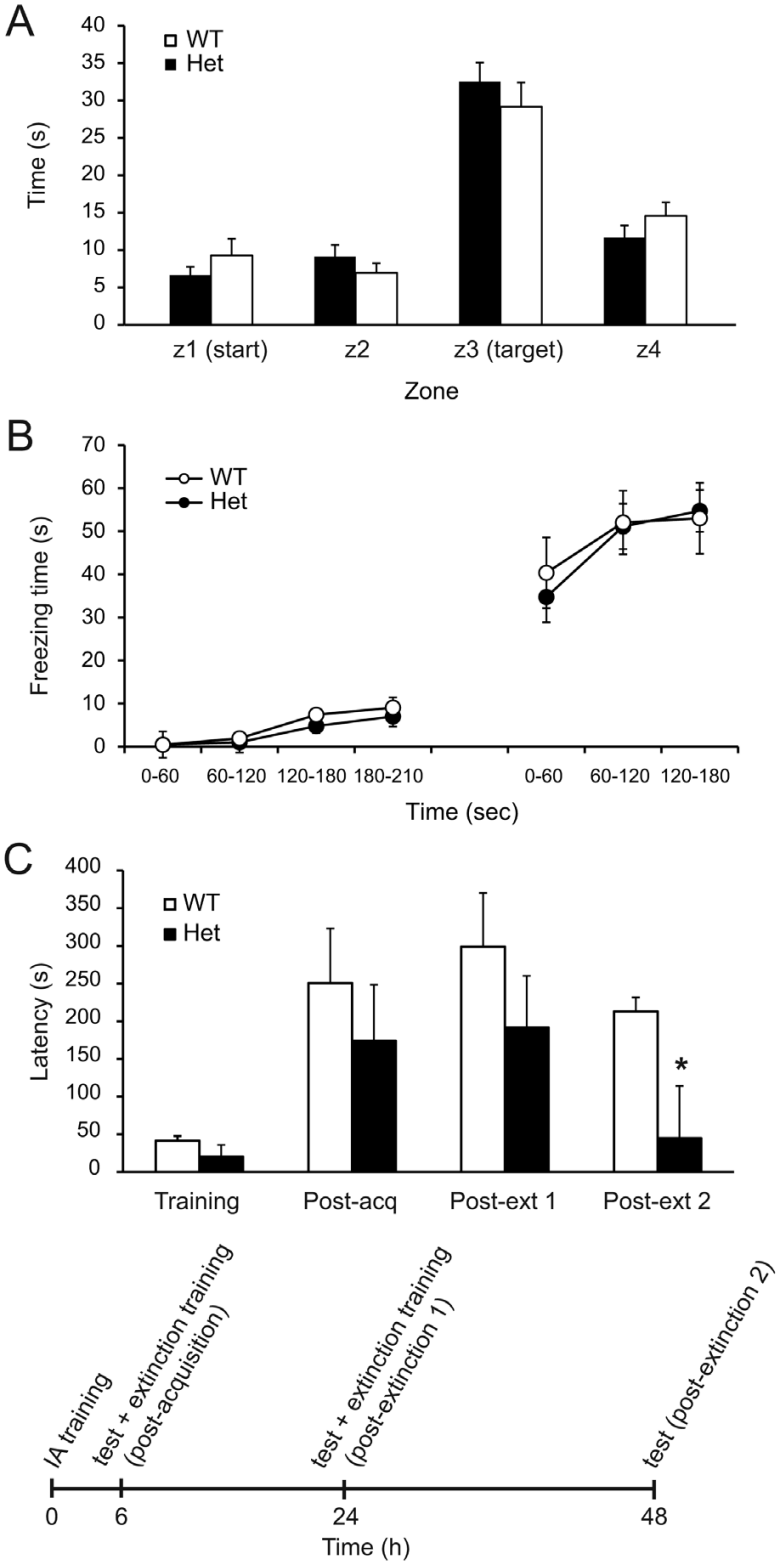
Normal learning and memory in Morris Water Maze and in fear conditioning but enhanced extinction of inhibitory avoidance in *Cyfip1* heterozygous mice. (**A**) Mice were tested using the Morris Water Maze. Time (s) to travel to the target platform was not significantly different between genotypes. (**B**) Mice were tested for fear conditioning, with mice receiving shocks at 120 and 180 seconds during training. Testing was performed 24 hours later, in the same test chamber, without footshock. (**C**) Inhibitory avoidance was measured by latency to enter the dark side of the box associated with prior shock. Extinction of inhibitory avoidance is enhanced in the heterozygotes. The lower panel shows the experimental design. WT, wildtype mice; Het, heterozygous mice; acq, acquisition; ext, extinction; IA, inhibitory avoidance. *, P = 0.027.

To examine the role of Cyfip1 in hippocampal dependent learning, we used a Y-maze (to detect alterations in working memory; data not shown) and Morris Water Maze (Fig. 5A) (to detect spatial learning and memory ability), which requires both an intact hippocampus and amygdala [30], [31], in *Cyfip1* heterozygote and wildtype mice. There were no differences between genotypes in these tests. Furthermore, we used a contextual fear paradigm and did not detect any changes between genotypes (Fig. 5B). All of these findings are similar to behavioral findings in *Fmr1* knockout mice. It has been shown, however, that *Fmr1* knockout mice show more rapid extinction in inhibitory avoidance testing [32], so it was of interest to perform inhibitory avoidance testing with the same paradigm. As has been described with *Fmr1* knockout mice, we observed a more rapid extinction timeline in the heterozygotes (at extinction 2, t-test, P = 0.027; Fig. 5C).

## Discussion

Based on the evidence for 15q11.2 deletion and duplications in psychiatric phenotypes and the presence of the FMRP-binding protein CYFIP1 in this interval, we carried out functional analyses of Cyfip1. Our data indicate that *Cyfip1* heterozygous mice exhibit reduced expression of Cyfip1, which is in turn associated with an enhancement of hippocampal mGluR-LTD, without affecting hippocampal LTP or even basal synaptic processes.

Protein synthesis is required for several different forms of synaptic plasticity, and control of protein synthesis is a critical mechanism for modulating long-term changes in neural circuits and resultant behavioral changes [33]. Protein synthesis is required for mGluR-LTD [23]. FMRP is an important regulator of translation in the brain and recently is has been shown that FMRP represses translation initiation (the rate limiting step in translation and hence an important target for regulation) via interaction with CYFIP1 [22]. These authors provide compelling evidence that CYFIP1 functions like other eukaryotic initiation factor (eIF) 4E-binding proteins (4E-BP), competing with eIF4G binding to eIF4E. Disrupting the eIF4E-eIF4G interaction inhibits translation as the bridge between the mRNA and the ribosomal pre-initiation complex is lost [33]. For canonical 4E-BP proteins, and perhaps for eIF4G, phosphorylation by an activated mTOR complex reverses the blockade on translation [33], a mechanism that may also occur with CYFIP1 [25]. In some studies, loss of FMRP leads to an increase in expression of target genes such as CamkII in synaptoneurosome, in steady state [25], [34]. We did not observe an increase in CamkII expression in our studies (data not shown). However as these effects are often subtle and difficult to measure, we cannot exclude an effect in basal levels of Fmrp-Cyfip1 targets in the absence of stimulation.

Here, we showed that incubation of slices with the mRNA translation inhibitor cycloheximide or the mTOR inhibitor rapamycin blocked mGluR-LTD in wildtype but not in heterozygous mice. The mTOR pathway plays a role in translation initiation and generation of translation elongation factors. The application of rapamycin prevents the phosphorylation of the translational regulator 4E-BP1 by mTOR. In its unphosphorylated state, 4E-BP1 remains bound to the translation initiation factor, eukaryotic initiation factor 4E (eIF4E), and the initiation of translation are inhibited.

In our experiments we observed that mGluR-LTD in *Cyfip1* heterozygous mice is insensitive to inhibition of protein synthesis demonstrating that the normal control of activity-regulated protein synthesis is lost in these mice. Previous work has demonstrated that mGluR-LTD was enhanced in *Fmr1* knockout mice and was unaffected by the presence of protein synthesis inhibitors [35]. The loss of the protein synthesis dependency of LTD in both these examples likely arises from a common mechanism, in which reduction of the levels of either member of a Cyfip1/Fmrp complex disrupts the baseline suppression of local translation in the synapse. Our findings also indicate that while mTOR plays a role in mGluR-LTD in wildtype mice, this mechanism is altered in *Cyfip1* heterozygous mice as we observed that rapamycin only reduced mGluR-LTD in studies with the wildtype animals. Altogether, our results support dysregulation of protein synthesis in the synapse and are consistent with studies in *Cyfip1* heterozygotes [25].

In our study, LTP induced by high frequency stimulation or threshold theta burst stimulation was unaltered in area CA1 in *Cyfip1* heterozygotes. LTP in CA1 induced by high frequency stimulation is also known to be unaffected in *Fmr1* knockout mice [36], [37], however, LTP elicited by threshold theta burst afferent stimulation is impaired in young adult *Fmr1* knockout mice [38] which is reversed by BDNF perfusion. Whether this is an age-specific effect or a difference between the two models remains to be determined.

How the alterations in hippocampal mGluR-LTD could contribute to the cognitive deficits is not known. Behavioral deficits in *Fmr1* knockout animals are typically quite subtle, however, extinction in a one-trial inhibitory avoidance paradigm, shown to be dependent on protein synthesis in the hippocampus, is enhance in *Fmr1* knockout animals [32]. Behavioral characterization of *Cyfip1* heterozygotes in our study also showed typical behaviours in many assays, including those assessing anxiety, social behaviours, and cognition, but more rapid extinction in inhibitory avoidance testing, similar to what has been described for *Fmr1* knockouts, supports a shared mechanism. However, because there is an effect of genetic background in some of the behavioural phenotypes in *Fmr1* knockout animals [39], [40] (see Table S2), the effect of genetic background must be considered in the modest behavioral changes in *Cyfip1*-deficient mice.

The development of a mouse model with a loss of a functional copy of *Cyfip1* provides an important resource to understand the role of this gene in psychiatric and neurological illnesses and in screening potential therapies. One potential intervention appears to be the targeting of mGluR with antagonists. It is of interest that use of two antagonists together produced a reversal of enhanced mGluR-LTD in *Cyfip1* heterozygous mice in our experiments, suggesting that a combined approach would be beneficial in both 15q11.2 CNV patients but also in FXS.

In summary, mice lacking one functional copy of *Cyfip1* show enhanced mGluR-LTD that is independent of protein synthesis and reversed by mGluR antagonists, as well as more rapid extinction in an inhibitory avoidance paradigm. These findings are identical to those observed in *Fmr1* knockout mice [24]. Note that our expression studies exclude an indirect effect of Cyfip1 depletion mediated through reduced expression of Fmrp, as Fmrp expression was normal. These observations indicate that gene dosage abnormalities of *CYFIP1* can alter synaptic plasticity and function, and supports shared mechanisms between FXS and phenotypes associated with the loss of a functional copy of *CYFIP1*. The partial loss of CYFIP1 expression, when considered together with the restricted regional expression of CYFIP1, especially as compared to the ubiquitous expression of CYFIP2 and FMRP, could explain why the behavioural consequences of CYFIP1-deficiency are not necessarily as severe as what is observed with loss of FMRP. Our studies are consistent with a model in which haploinsufficiency of *CYFIP1* leads to an intermediate phenotype that, in the context of additional factors, can results in divers neuropsychiatric conditions.

## Materials and Methods

### Generation of Mice with a Disruption of the *Cyfip1* Gene

All animal procedures were approved by the IACUC at Mount Sinai School of Medicine and the Bronx VA Medical Center.

Mice were developed from an Omnibank (Lexicon) embryonic stem (ES) cell line, with *Cyfip1* targeted by mutagenesis with a gene trap insertional vector. Briefly, we identified an ES clone that has a trapping cassette inserted into intron 1 of the *Cyfip1* gene (note that the start ATG is in exon 2). A mouse line was established from the ES cells in the 129SvEvBrd strain, subsequently backcrossed to C57Bl/6Tac.

### Analysis of Developmental Milestones

All testing was done blind to genotype. We made use of a systematic approach to assess development in the mice, following our prior approach [41], as well as a recent detailed protocol [42]. We tested cohorts beginning at 3 days in 3-day increments until the animals were 27 days old; the tester kept track of individual pups by marking their tails with a non-toxic, low odor marker. A total of 5 litters were assessed. Each pup was observed for physical development and tested on a number of reflexes. To assess physical development, body weight was measured, while hallmarks including fur development, incisor eruption, eye opening and detachment of pinnae were observed and noted. Motor development and reflexes were monitored by appearance and/or disappearance of the righting reflex, crossed extensor reflex, and grasp reflex, and by performance in negative geotaxis, level screen test, vertical screen test, and bar holding, drawn from standard SHIRPA descriptions [43]. Sensory and motor coordination was monitored by the appearance of cliff avoidance, forelimb placing, vibrissa placing, visual placing, auditory startle, tactile startle, and toe pinch. Fear-induced freezing was measured after the pup was placed in a 100-ml beaker and dropped by inverting the beaker.

### Behavioral Analysis

We prepared cohorts of 28 male animals (13 wildtype and 15 heterozygotes) from 6 litters from wild type x heterozygote matings. Behavioral studies were conducted at the Rat and Mouse Phenotyping Shared Research Facility at Mount Sinai School of Medicine. Mice were transferred to the Facility at 2 months of age, acclimated for 2 months, and went through a test battery starting at 4 months of age. Most procedures have been described previously [44], [45]. The order of behavioral testing was general observation, open-field, light dark transition, elevated zero maze, social interactions, Y-maze, Morris Water Maze, conditioned fear testing, inhibitory avoidance, and PPI. Morris Water Maze trials were run in a 48" plastic pool. On the first day of the 2-week procedure, test animals were habituated to the pool in a 5 min trial with the platform visible and accessible, extending just out of the water. Test animals were placed in the quadrant opposite the platform, and, as on all subsequent trials, facing into the wall. In the subsequent 8 days of training trials each subject received 4 1-minute trials per day, 10–15 minutes apart. Training days fell in two groups of 4 consecutive days, 2 days apart. In all training days, subjects began one trial in each quadrant, counterclockwise from a starting quadrant that also shifted clockwise one step each day. Each subject’s total elapsed time to find the platform was recorded, and the trial ended if the subject remained on the platform for 5 seconds or more. On the final day, the 9th (probe) day, the platform was removed entirely and subjects given a single 1 min trial starting from the quadrant opposite the original platform location. In this probe trial, latency to reach the target square, time spent in the target quadrant, and time spent in the target square were quantified along with time spent in all other quadrants and corresponding target areas.

Inhibitory avoidance was performed following the protocol published previously [32] except we used longer cut off times (180 s instead of 120 s) during our initial training phase. We tested at 6 hrs, 24 hours and 48 hours after initial training. We used an inhibitory avoidance box from San Diego Instruments. For training, subjects spend 30 s in dark chamber and were then moved to the start box in the light chamber for 90 s of habituation (gate closed). When the gate opened the light remained on and latency to cross through to dark side was measured (baseline). Once the subject crossed into dark chamber, the gate was closed and the subject receives 2 s of 0.5 mA footshock. After 15 s, the subject was returned to its home cage. Animals with baseline cross-through latencies greater than 180 s were excluded. Six hours later, subjects were tested on retention. After 90 s in light side with gate closed, the gate was opened, and cross-through latency was recorded (with a 540 s cutoff). For the extinction phase subjects were allowed to freely explore the dark chamber for 200 s, with no footshock, before being returned to their home cages. 24 hours after training, post-extinction 1, was carried out in a manner identical to the retention, and, again at 48 hours after training, post-extinction 2 was carried out, also identical to retention, minus 200 s exploration after cross-through.

### Protein Analysis

We prepared synaptoneurosomes from whole cortex of animals at 4 weeks of age for protein analysis [46], using 4 pairs of heterozygotes and littermate controls for the analyses. Equal amount of proteins were subjected to SDS PAGE, followed by quantitative immunoblotting using a Li-COR system (Li-COR). Images were quantitated by Image-J and the intensity of bands normalized to a reference actin signal for each lane. Antibodies used were anti-Sra-1 (Synaptic systems), anti-Fmrp (Millipore), anti-CamKII (Millipore), and anti-actin (Sigma). The anti-Sra-1 antibody gives only one band in brain extracts as shown in Figure 1.

### Quantitative PCR Analysis

Quantitative PCR (qPCR) using the Universal Probe Library system (Roche) was performed as described previously [47]. We designed primers with ProbeFinder (Roche), making use of multiple reference genes for normalization, with data analysis carried out with qBase software. Control genes used were Actb, Gusb, 18S rRNA, and Rpl. Six wild type and six heterozygous male animals (5 weeks old) were used. RNA was prepared from dissected prefrontal cortex using RNAeasy kit (Qiagen), and used for making total cDNA using random primers, and 25 ng of total cDNA was used for the qPCR.

### Hippocampal Slice Electrophysiology

Hippocampal slices (350 µm) were prepared from 4–6 week old heterozygous mice and their wildtype littermate controls. Slices were perfused with Ringer’s solution containing (in mM): NaCl, 125.0; KCl, 2.5; MgSO_4_, 1.3; NaH_2_PO_4_, 1.0; NaHCO_3_, 26.2; CaCl_2_, 2.5; glucose, 11.0. The Ringer’s solution was bubbled with 95% O_2_/5% CO_2_, at 32°C, during extracellular recordings (electrode solution: 3 M NaCl). Slices were maintained for 1 hr prior to establishment of a baseline of field excitatory postsynaptic potentials (fEPSPs) recorded from stratum radiatum in area CA1, evoked by stimulation of the Schaffer collateral-commissural afferents (100 µs pulses every 30 s) with bipolar tungsten electrodes placed into area CA3 [48]. Test stimulus intensity was adjusted to obtain fEPSPs with amplitudes that were one-half of the maximal response. The EPSP initial slope (mV/ms) was determined from the average waveform of four consecutive responses.

Cycloheximide (60 µM, Sigma), (S)-3,5-dihydroxyphenylglycine (DHPG, 50 µM, Sigma), 2-methyl-6-phenylethynyl-pyridine (MPEP, 10 µM, Tocris), LY367385 (100 µM, Tocris), or rapamycin (20 nM, Enzo Life Sciences) were bath-applied for durations indicated in the figure legends. All experiments were performed in the presence of 100 µM 2-amino-5-phosphopentanoic acid (AP5).

Paired-pulse responses were measured with an interstimulus interval (ISI) of 50 ms, and were expressed as the ratio of the average responses to the second stimulation pulse (FP2) to the first stimulation pulse (FP1). Long-term potentiation (LTP) was induced by either a high-frequency stimulus (four trains of 100 Hz, 1 s stimulation separated by 5 min), threshold levels of theta-burst stimulation (TBS) (5 bursts of four pulses at 100 Hz separated by 200 ms, [38], or a single 100 Hz stimulation. To induce an mGluR-dependent long-term depression (LTD), Schaffer collaterals were stimulated by a paired-pulse low-frequency stimulation (PP-LFS, 1 Hz for 20 min; 50 ms interstimulus interval [23]. DHPG-induced LTD was also used where indicated.

### Data Analysis

Data are expressed as mean ± SD, and statistical analyses were performed using either a two-way repeated-measures ANOVA, ranged from post-LTP or LTD-inducing stimuli onward until the end of recording, or Student’s t-test, where P<0.05 was considered significant. N’s indicate number of slices (1–3 slices from 3–6 mice per group).

## Supporting Information

Table S1 **Anxiety-related behavioral measures in** ***Cyfip1*** **heterozygous mice and wildtype littermates.** In the measures for open field, light dark transition and elevated zero maze tests, there were no differences between wildtype and *Cyfip1* heterozygous animals.
(DOC)

Table S2 **Comparison of behavioral phenotypes from** ***Cyfip1*** **heterozygous mice and** ***Fmr1*** **knockout mice on two different backgrounds.** Results of our behavioural assays in *Cyfip1* heterozygous mice are compared to *Fmr1* knockout mice, based on published data in *Fmr1* knockout mice on different backgrounds.
(DOC)
