# Supplementary material for: Haploinsufficiency of Cyfip1 Produces Fragile X-Like Phenotypes in Mice
Source: PLoS One. 2012 Aug 10;7(8):e42422. doi: 10.1371/journal.pone.0042422 (PMC3416859; doi:10.1371/journal.pone.0042422)
Supplement: Table S1 — Anxiety-related behavioral measures in Cyfip1 heterozygous mice and wildtype littermates. In the measures for open field, light dark transition and elevated zero maze tests, there were no differences between wildtype and Cyfip1 heterozygous animals. (DOC) [file pone.0042422.s001.doc]

| **Test** |  | Het (n=15)  Mean +/- sd | WT (n=13)  Mean +/- sd |
| --- | --- | --- | --- |
| **1-hour activity** | Total distance (cm) | 3451 +/-1965 | 1965 +/-2402 |
|  | Move time (s) | 408 +/-209 | 500 +/-265 |
|  | Rears | 131 +/-130 | 239 +/-168 |
|  | Total revolutions | 24.6 +/-15.5 | 29.8 +/-16.0 |
|  | Percent clockwise revs | 53.6 +/-29.8 | 53.1 +/-19.7 |
| **Open field (10m)** | Center entries | 20.7 +/-18.7 | 25.4 +/-18.8 |
|  | Center time (s) | 36.2 +/-31.0 | 40.7 +/-35.4 |
| **Elevated zero** | Open time, day 1 (s) | 50.7 +/-34.2 | 34.5 +/-15.6 |
|  | Open time, day 2 (s) | 90.2 +/-38.4 | 72.4 +/-34.0 |
|  | Open latency, day 1 (s) | 35.4 +/-76.0 | 28.0 +/-38.6 |
|  | Open latency, day 2 (s) | 24.1 +/-76.5 | 7.64 +/-9.37 |
|  | Cross latency, day 1 (s) | 57.7 +/-84.1 | 72.4 +/-96.0 |
|  | Cross latency, day 2 (s) | 52.6 +/-88.9 | 110.7 +/-123.2 |
| **Light/dark emergence** | Light edge latency (s) | 188.4 +/-257.5 | 176.3 +/-242.2 |
|  | Light center latency (s) | 235.0 +/-248.4 | 223.3 +/-261.9 |
|  | Total light time (s) | 98.0 +/-91.2 | 72.0 +/-56.0 |
|  | Total light entries | 27.0 +/-23.7 | 22.8 +/-16.8 |
