# Supplementary material for: Haploinsufficiency of Cyfip1 Produces Fragile X-Like Phenotypes in Mice
Source: PLoS One. 2012 Aug 10;7(8):e42422. doi: 10.1371/journal.pone.0042422 (PMC3416859; doi:10.1371/journal.pone.0042422)
Supplement: Table S2 — Comparison of behavioral phenotypes from Cyfip1 heterozygous mice and Fmr1 knockout mice on two different backgrounds. Results of our behavioural assays in Cyfip1 heterozygous mice are compared to Fmr1 knockout mice, based on published data in Fmr1 knockout mice on different backgrounds. (DOC) [file pone.0042422.s002.doc]

|  | **Fmr1 B6** | **Fmr1 FVB** | **Cyfip1 B6** |
| --- | --- | --- | --- |
| **Inhibitory Avoidance** | Normal ^1,2^ | Normal ^3^ | Normal |
| **Inhibitory Avoidance Extinction** | Enhanced ^2^ |  | Enhanced |
| **Open field** | Normal ^4^ | Normal ^5^ | Normal |
| **Rotarod** | Normal ^6^ |  | Normal |
| **Light-Dark Transition** | Increased ^1,6,7^ | Normal ^7^ | Normal |
| **Conditioned Fear** | Normal ^6,7,8,9^ | Normal ^8^ | Normal |
| **Morris Water Maze** | Mild impairment ^1^ | Mild  impairment ^8^ | Normal |
| **Elevated Zero Maze** | Normal ^4,5^ | Normal ^10^ | Normal |
| **PPI** | Enhanced ^5,11^ | Subtle change^12^ | Normal |
| **Auditory Startle** | Impaired-at high  Intensities ^5,11^ | Mild  Impairment ^3,12^ | Normal |
| **Social Interaction** | Impaired ^13, 14^ | Impaired ^13^ | Normal |

^1^ The Dutch-Belgian Fragile X Consortium (1994) *Fmr1* knockout mice: a model to study fragile X mental retardation. *Cell* 78, 23–33.

^2^ Dölen G., Osterweil E., Rao B.S., Smith G.B., Auerbach B.D., Chattarji S., Bear M.F. (2007) Correction of fragile X syndrome in mice. *Neuron* 20;56(6):955-62.

^3^ Qin M., Kang J., Smith C.B. (2005) A null mutation for Fmr1 in female mice: effects on regional cerebral metabolic rate for glucose and relationship to behavior. *Neuroscience*.135:999–1009.

^4^ Mineur Y.S., Sluyter F., de Wit S., Oostra B.A., Crusio W.E. (2002) Behavioral and neuroanatomical characterization of the *Fmr1* knockout mouse. *Hippocampus* 12, 39–46.

^5^ Nielsen D.M., Derber W.J., McClellan D.A., Crnic L.S. (2002) Alterations in the auditory startle response in Fmr1 targeted mutant mouse models of fragile X syndrome. *Brain Res.* 8;927(1):8-17.

^6^ Peier A.M., McIlwain K.L., Kenneson A., Warren S.T., Paylor R., and Nelson D.L. (2000) (Over)correction of FMR1 deficiency with YAC transgenics: behavioral and physical features. *Hum. Mol. Genet.* 9, 1145–1159.

^7^ Spencer C.M., Alekseyenko O., Hamilton S.M., Thomas A.M., Serysheva E., Yuva-Paylor L.A., Paylor R. (2011) Modifying behavioral phenotypes in Fmr1KO mice: genetic background differences reveal autistic-like responses. *Autism Res*. Feb;4(1):40-56.

^8^ Dobkin C., Rabe A., Dumas R., El Idrissi A., Haubenstock H., and Brown W.T. (2000) *Fmr1* knockout mouse has a distinctive strain-specific learning impairment. *Neuroscience* 100, 423–429.

^9^ Van Dam D., D'Hooge R., Haube E., Reyniers, E., Gantois I., Bakker C.E., Oostra B.A., Kooy R.F., and De Deyn P.P. (2000) Spatial learning, contextual fear conditioning and conditioned emotional response in *Fmr1* knockout mice. *Behav. Brain Res.* 117, 127–136.

^10^ Zhao M.G., Toyoda H., Ko S.W., Ding H.K., Wu L.J., and Zhuo M. (2005) Deficits in trace fear memory and long-term potentiation in a mouse model for fragile X syndrome. *J. Neurosci.* 25, 7385–7392.

^11^ Frankland P.W., Wang Y., Rosner B., Shimizu T., Balleine B.W., Dykens E.M., Ornitz E.M., and Silva A.J. (2004) Sensorimotor gating abnormalities in young males with fragile X syndrome and *Fmr1*-knockout mice. *Mol. Psychiatry* **9**, 417–425.

^12^ Chen L. and Toth M. (2001) Fragile X mice develop sensory hyperreactivity to auditory stimuli. *Neuroscience* **103**, 1043–1050.

^13^ Moy S.S., Nadler J.J., Young N.B., Nonneman R.J., Grossman A.W., Murphy D.L., et al. (2009) Social approach in genetically engineered mouse lines relevant to autism. *Genes Brain and Behavior*;8(2):129–142.

^14^ Pietropaolo S., Guilleminot A., Martin B., D’Amato F.R., Crusio W.E. (2011) Genetic-Background Modulation of Core and Variable Autistic-Like Symptoms in Fmr1 Knock-Out Mice. *PLoS ONE* 6(2): e17073.
